# Perfusion Phantom: An Efficient and Reproducible Method to Simulate Myocardial First-Pass Perfusion Measurements with Cardiovascular Magnetic Resonance

**DOI:** 10.1002/mrm.24299

**Published:** 2012-04-24

**Authors:** Amedeo Chiribiri, Andreas Schuster, Masaki Ishida, Gilion Hautvast, Niloufar Zarinabad, Geraint Morton, James Otton, Sven Plein, Marcel Breeuwer, Philip Batchelor, Tobias Schaeffter, Eike Nagel

**Affiliations:** 1 Division of Imaging Sciences, King's College London BHF Centre of Excellence, NIHR Biomedical Research Centre and Wellcome Trust and EPSRC Medical Engineering Centre at Guy's and St. Thomas' NHS Foundation Trust, The Rayne InstituteLondon, United Kingdom; 2 Philips Healthcare, Imaging Systems—MR, BestThe Netherlands; 3 Division of Cardiovascular and Neuronal Remodelling, University of LeedsLeeds, United Kingdom; 4 Department of Biomedical Engineering, Biomedical Image Analysis, Eindhoven University of TechnologyEindhoven, The Netherlands

**Keywords:** first-pass perfusion MR, phantom, gadolinium, 3T, heart

## Abstract

The aim of this article is to describe a novel hardware perfusion phantom that simulates myocardial first-pass perfusion allowing comparisons between different MR techniques and validation of the results against a true gold standard. MR perfusion images were acquired at different myocardial perfusion rates and variable doses of gadolinium and cardiac output. The system proved to be sensitive to controlled variations of myocardial perfusion rate, contrast agent dose, and cardiac output. It produced distinct signal intensity curves for perfusion rates ranging from 1 to 10 mL/mL/min. Quantification of myocardial blood flow by signal deconvolution techniques provided accurate measurements of perfusion. The phantom also proved to be very reproducible between different sessions and different operators. This novel hardware perfusion phantom system allows reliable, reproducible, and efficient simulation of myocardial first-pass MR perfusion. Direct comparison between the results of image-based quantification and reference values of flow and myocardial perfusion will allow development and validation of accurate quantification methods. **Magn Reson Med, 2013. © 2012 Wiley Periodicals, Inc.**

## INTRODUCTION

First-pass myocardial MR perfusion has become a reliable tool for the diagnosis of myocardial ischemia (1). Although myocardial perfusion MR images are usually evaluated by visual assessment (2) or by semiquantitative approaches (3), quantitative analysis and absolute quantification have also been described and may permit a more accurate assessment of patients with heart disease, particularly those with three-vessel coronary artery disease (4). Quantitative analysis was initially proposed more than a decade ago and has achieved a recognized role as an investigational tool. However, it has not been adopted into clinical routine thus far. One of the main reasons is the lack of standardization of the analysis methods (5), which is partly due to the lack of a gold standard for validation of the results. Novel techniques are currently developed using combinations of numerical simulations, animal studies, and human trials (4, 5).The aim of this study is to describe and validate a novel MR perfusion phantom hardware capable of simulating the process of first-pass perfusion in a highly controllable and reproducible way and thus provide true physical validation of quantitative perfusion methodologies.

## METHODS

**FIG. 1:**
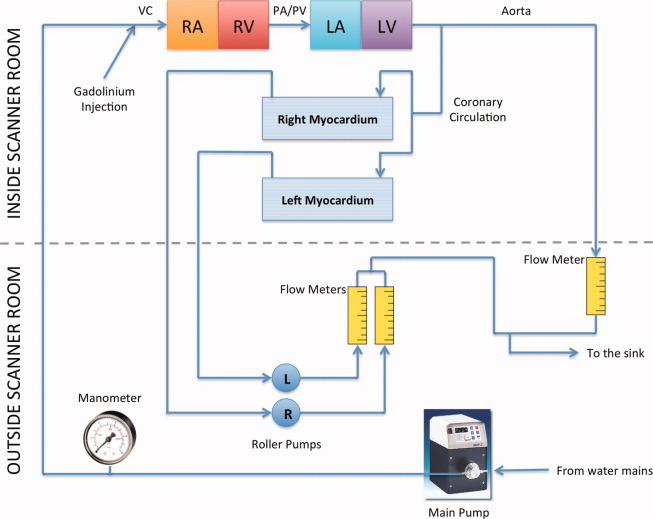
Schematic representation of the system. Three main units constitute the perfusion phantom: the main pump and the control unit—located outside the MR room—and the MR compatible unit (the phantom) in the bore of the scanner. The main pump generates the water flow across the phantom. Just before the VC, a three-way tap allows the injection of the contrast agent into the circuit using a clinical power injector. The flow travels across the cardiac chambers and the thoracic vessels to reach the aorta, where a portion of the flow is directed toward the right and left myocardial compartment. The water flow from the aorta after the take off of the coronary circulation is then directed back outside the scanner room to the control unit where it is continuously measured by means of a vertical flow meter. The flow from the right and left myocardial compartments is returned in two separate pipes to roller pumps—part of the control unit—that allow fine regulation of the flow across each compartment. At the outlet of each roller pump, a vertical flow meter continuously measures the flow across each myocardium. LA, left atrium; LV, left ventricle; PA, pulmonary artery; PV, pulmonary vein; RA, right atrium; RV, right ventricle. [Color figure can be viewed in the online issue, which is available at wileyonlinelibrary.com.]

**FIG. 2:**
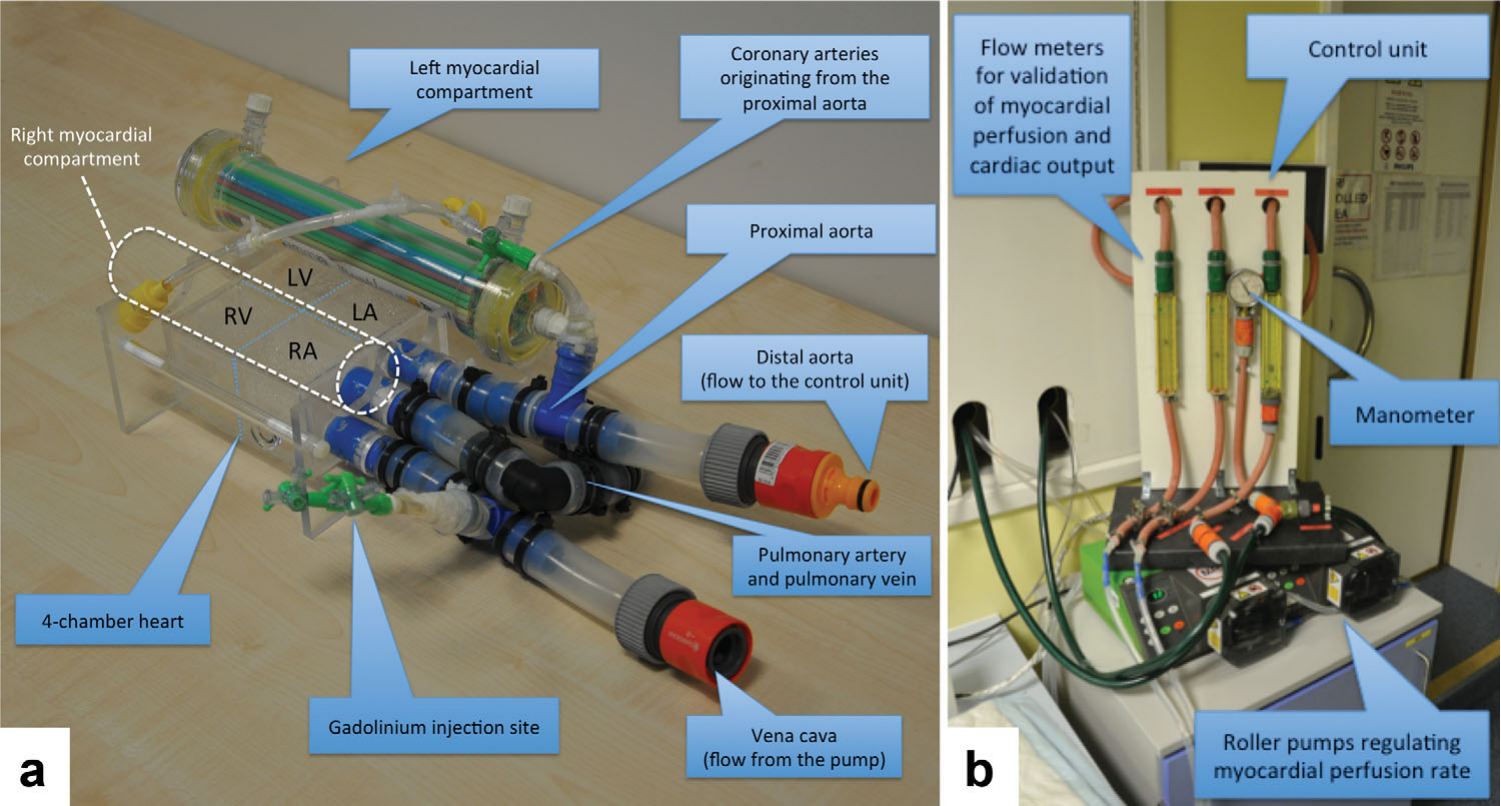
**a**: Picture of the perfusion phantom. The right myocardial compartment was removed and replaced with the dotted graph to allow visualization of the four-chamber heart located below. **b**: Control unit and roller pumps. The unit provides fine control of myocardial perfusion flow and precise measurement of cardiac output, maximum pressure in the circuit, and myocardial perfusion. RA, right atrium; RV, right ventricle; LA, left atrium; LV, left ventricle. [Color figure can be viewed in the online issue, which is available at wileyonlinelibrary.com.]

The phantom was designed to simulate dynamic of first-pass myocardial MR perfusion after the injection of a bolus of a gadolinium-based contrast agent. The system is made up of three main parts: the main pump generating water flow in the circuits located outside the MR room, the MR-compatible unit (the phantom) located in the scanner, and the control unit located outside the MR scanner room (Figs. 1 and 2).

### The Main Pump

The main pump maintains the water flow across the phantom and is located outside the MR room (Fig. 1). Various pumps producing continuous or pulsatile flow can be fitted to the system. Alternatively, it can be driven by water pressure from a water tap, as performed in our laboratory in some preliminary experiments (data not shown). Furthermore, the system can be configured as an open or a closed circuit. In the open circuit configuration (Fig. 1), the system is continuously supplied with clean water from the water mains and the volume of water and gadolinium flowing back from the phantom is discarded. In this setup, the background signal intensity (SI) values return to baseline in 60–180 s (depending on the myocardial perfusion rate) in preparation for subsequent gadolinium injections. In the closed circuit configuration, the reflowing water is recycled back through the system, with the effect of increasing background signal as the concentration of contrast agent increases in the circuit. The closed circuit configuration also allows modification of the recirculating perfusate. All the data presented in this manuscript were obtained with the open circuit setup, driven by a constant flow pump (model ISM 405A, Ismatec, Glattbrugg, Switzerland—pump-head model 201-000, Micropump, Vancouver, WA). By adjusting the speed of the main pump, the cardiac output of the phantom can be varied between 2 and 11 L/min. At a simulated heart rate of 60 beats per minute, a cardiac output of 4 L/min corresponds to a stroke volume of 67 mL. As a reference, the same cardiac output in a 60 kg/170 cm patient (body surface area of 1.68 m^2^) would be equivalent to a cardiac index ranging from 1.2 to 6.6 L/min/m^2^.

### The Phantom

To reproduce the dilution of the contrast bolus and its mixing with blood that occurs in the large thoracic vessels and in the heart, the phantom was designed to resemble the anatomy of the thoracic circulation and of the heart of a 60 kg human subject (Figs. 1 and 2a). The inner blood volume of each section was sized to resemble physiological size as closely as possible (Table 1). Moreover, the body-weight-adjusted volume of contrast agent administered in each experiment was calculated for this 60 kg value. For the sake of simplicity, in this article, we will refer to each segment of the phantom by the name of the anatomical structure it represents (their technical specifications are reported in Table 1).

**Table 1:** Components of the Perfusion Phantom and Their Characteristics

Section	Subsection	Size	Material
Heart	Right and left atrium	105 mL	Poly(methyl methacrylate) box
	Right and left ventricle	120 mL	Poly(methyl methacrylate) box
Vena cava	–	1.6-cm diameter × 13-cm length	Silicone tube
		Inner volume 26 mL	
Pulmonary artery/vein	–	1.6-cm diameter × 44-cm length	Silicone tube
		Inner volume 88 mL	
Aorta (before coronary arteries)	–	1.6-cm diameter × 18-cm length	Silicone tube
		Inner volume 36 mL	
Coronary arteries	–	0.5-cm diameter × 30-cm length	Polyvinyl chloride (PVC) tube
		Inner volume 5.8 mL	
Myocardium	–	2-cm radius; 12.6 cm^2^ section	Polypropylene (PP) tubes in a poly(methyl methacrylate) box

The core of the system is a four-chamber heart and two cylinders representing the myocardial compartments (Figs. 1 and 2a). The left ventricle (LV) and the right ventricle have a volume of 120 mL each. The right atrium (RA) and the left atrium have a volume of 105 mL each.

The heart receives a positive pressure water flow from a pipe connecting the vena cava (VC) to the main pump and to the control unit. Just before the VC (15 cm before the RA), a three-way stopcock allows direct injection of contrast agent into the water flowing in the circuit. This operation was performed by a clinical power injector (Spectris Solaris, MEDRAD Inc., Warrendale, PA), which allows contrast to be administered in the same way as it is for usual clinical protocols.

After the injection, the bolus of contrast agent travels in the water through the chambers and vessels and it is progressively mixed and diluted in water. Similar to the fragmentation of the bolus of gadolinium observed in vivo, the system generates the arterial input function (AIF) measured in the proximal aorta that can be used for quantification of myocardial perfusion by means of signal deconvolution techniques.

The bolus flows through the RA and the right ventricle, which is connected to the left atrium by a silicone tube (Fig. 2a). After the LV, the flow enters the aortic vessel, where a small polyvinyl chloride pipe gives origin to the coronary circulation that connects to the right and the left myocardial compartments. A defined volume of the flow (precisely regulated and measured by the control unit) enters both the right and the left myocardial compartments after the bifurcation of the polyvinyl chloride pipe (see below for details about flow/perfusion gold standard measurements).

**FIG. 3:**
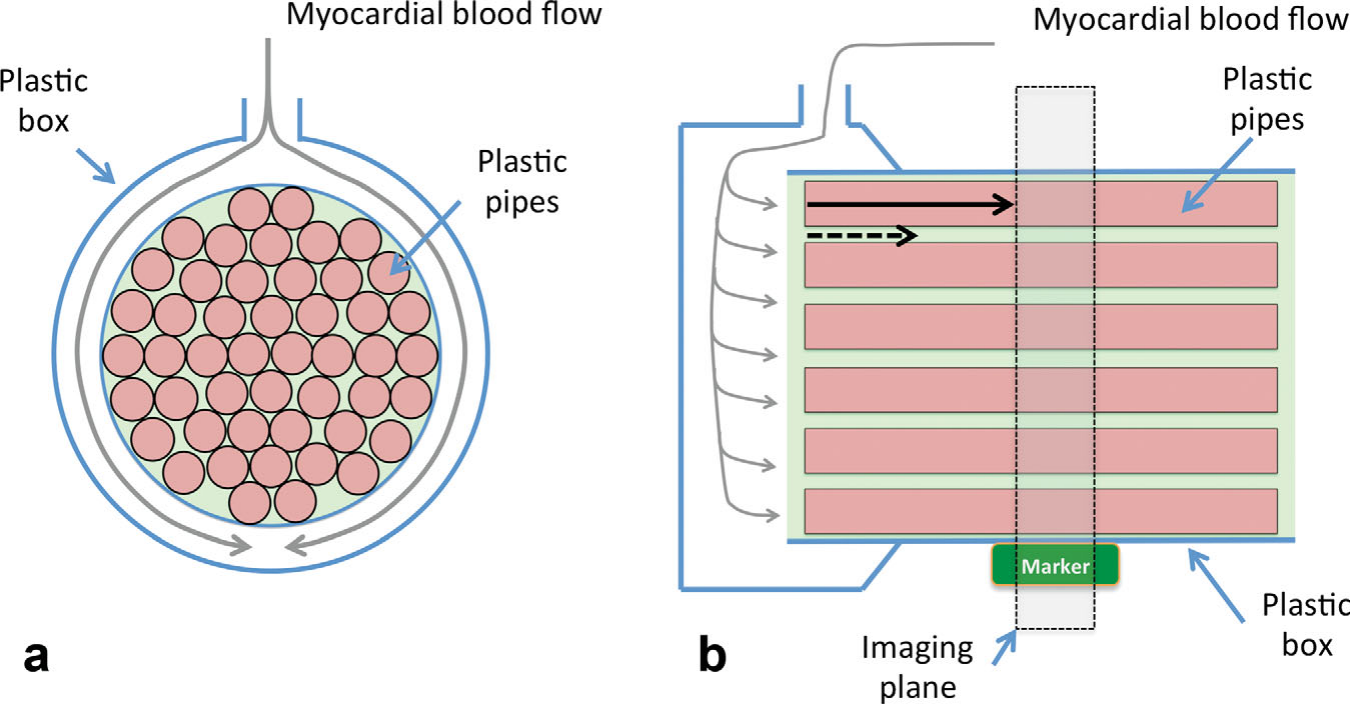
Schematic representation of the myocardial compartments. **a**: Short-axis view at the level of the myocardial flow inlet, represented by a lateral opening in the compartment. The simulated myocardial blood flow distributes to a circular space surrounding the inlet of the pipes first and then (**b**) enters the pipes. These are 124 parallel polypropylene pipes (48 shown in this scheme). Myocardial SI curves are generated in the imaging plane during first pass of the bolus of contrast agent, which follows two different pathways: inside the pipes (solid black arrow) and with slower speed in the space between one pipe and the others (dotted arrow). Both components generate the dynamic first-pass signal intensity upslope. The imaging plane is located at the level of a marker that identifies a myocardial distribution volume of 45 mL. This value allows the calculation of the gold standard perfusion rate from perfusion flow measurements. Representation not to scale. [Color figure can be viewed in the online issue, which is available at wileyonlinelibrary.com.]

Both myocardial compartments are plastic cylinders of 4-cm diameter containing 124 pipes with a thin (0.1 mm) polypropylene wall and with a diameter of 3 mm. Coronary blood flow enters the myocardial compartments from a lateral inlet, ensuring an even distribution of the perfusion flow during first pass across the section of the cylinder. The mechanism by which physiological first-pass SI curves for the myocardial compartments are generated and a detailed description of the myocardial compartments are explained in detail in the legend of Fig. 3. Two independent pipes collect the water flow from the myocardial compartments and return it independently to the control unit (Figs. 1 and 2b), where flow rates can be accurately measured and controlled in the range of 0.035–0.45 L/min.

**FIG. 4:**
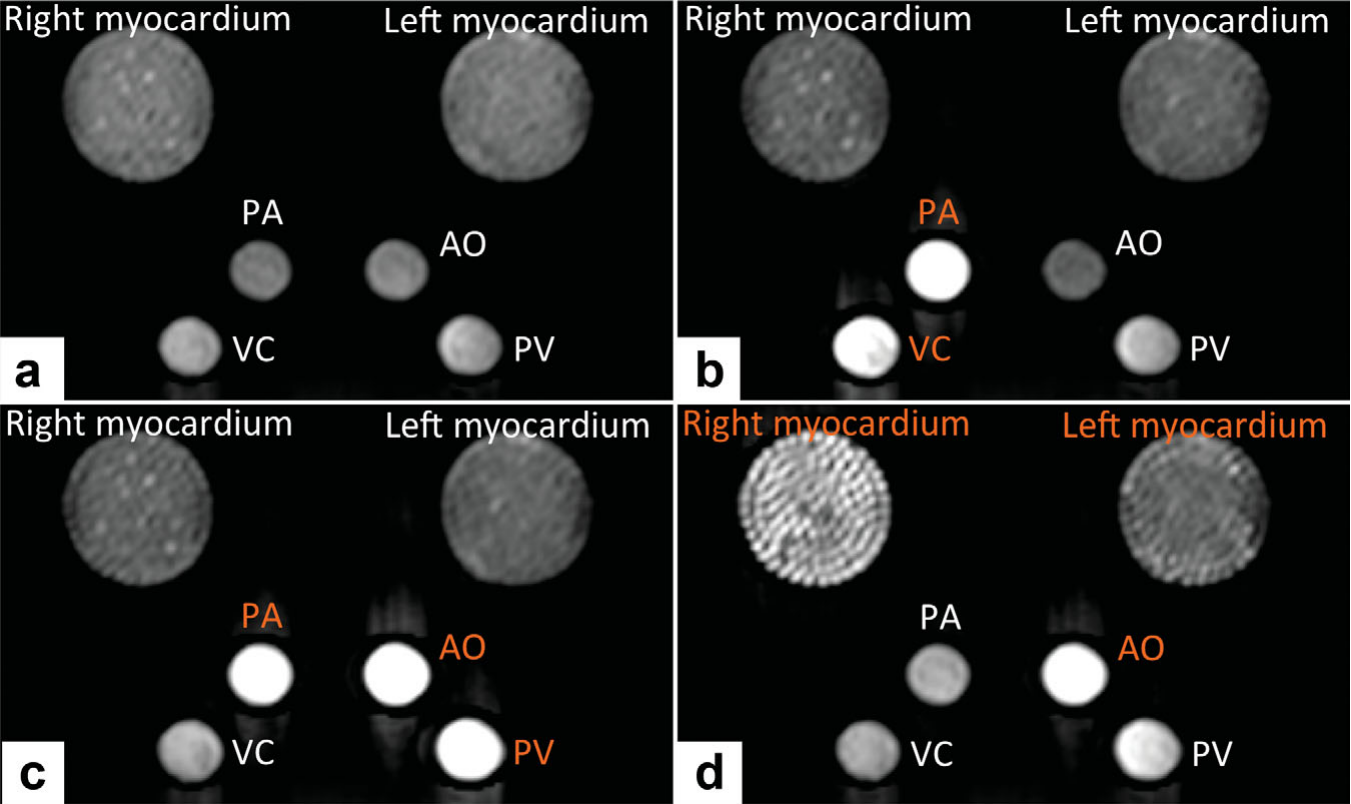
Example of consecutive dynamics obtained from the perfusion phantom. **a**: Baseline image, before contrast injection. **b**: Early image, with SI increase in the VC and pulmonary artery. **c**: SI increase in the pulmonary artery, pulmonary vein, and aorta. **d**: SI increase in the aorta, right myocardial compartment (perfusion rate 10 mL/mL/min) and initial signal increase in the left myocardial compartment (5 mL/mL/min). [Color figure can be viewed in the online issue, which is available at wileyonlinelibrary.com.]

To relate the gold standard flow rate across the myocardial compartments with the measured perfusion rate, the myocardial compartments were titrated to define the distribution volume of the contrast agent during first pass. The distribution volume is the water effectively modifying the distribution of the contrast agent and the characteristics of the SI curves during first pass and was defined as the volume of water comprised between the point where the aortic AIF is sampled (just before the take off of the coronary circulation) and the myocardial volume preceding and including the imaging plane. Because of the complex geometry of this section, the position of the imaging plane was defined by weighing each myocardial compartment (kept in vertical position) and its coronary vessel on a precision scale and adding 45 g of distilled water, corresponding to 45 mL of volume. To facilitate the identification of the correct geometry during scanning, the level corresponding to the imaging plane was marked on the outer surface by a multimodality marker (Multi Modality Marker 3003, IZI Medical Products, Owings Mills, MD; Fig. 3). The plastic pipes do not have any filtration function and do not constitute a separate compartment for the diffusion of the contrast agent within the myocardial space. Therefore, the myocardial space acts as a single compartment for the distribution of gadolinium. Referred to the distribution volume of 45 mL, flow rates ranging from 0.035 to 0.45 L/min correspond to perfusion rates ranging from 0.8 to 10 mL of perfusate per milliliter of distribution volume per minute [mL/mL/min].The phantom itself is contained in a plastic box and can be used with any surface array coil used for parallel cardiac imaging. The design of the phantom allows the acquisition of the MR images of the aorta and the myocardial compartments in the same imaging plane (Fig. 4).

### The Control Unit

The control unit (Figs. 1 and 2b) is located outside the MR room and is designed to allow precise measurements of flow in each compartment of the phantom (gold standard reference for perfusion and cardiac output) and fine control of the functional parameters of the system. The control unit receives the forward flow from the main pump and measures the maximum pressure in the water circuit by means of an aneroid manometer (Model EN837; Nuova FIMA, Novara, Italy; Figs. 1 and 2b). This permits prompt identification of any leakages (pressure drops to zero) or obstructions (pressure rises above 50 kPa). During normal operation, the maximum pressure in the circuit reaches approximately 25 kPa for a forward flow of 3 L/min, and 40 kPa for 4 L/min. After passing the manometer, the forward flow continues toward the VC of the phantom. The control unit receives the return flow from the phantom via three independent pipes (distal aortic flow; right and left myocardial compartment). The distal aortic flow is measured by a vertical flow meter (model S.800002, Parker, RS Components, Corby, United Kingdom) before being discarded or recirculated through the system, depending on whether the water circuit is in an open or closed configuration.

The return flow from each myocardial compartment is brought back independently to the control unit where two roller pumps (Model U505, Watson-Marlow, Falmonth, United Kingdom) regulate precisely and independently the perfusion rate in each myocardial compartment. The roller pumps were positioned distal to the myocardial compartments to minimize the dead space between the ascending aorta and the myocardial compartments. Positioning the pumps between the ascending aorta and the myocardium might interfere with the dilution of the contrast agent and therefore affect quantitative perfusion measurements. At the exit of the roller pumps, the flow rate in the right and left myocardial compartment lines is measured by two vertical flow meters (model S.800003, Parker, RS Components, Corby, United Kingdom).

### MR Methods

All data were acquired on a 3T Philips Achieva TX system, equipped with a 32-channel cardiac phased array receiver coil (Philips, Best, The Netherlands). Perfusion data were acquired in a transverse geometry, visualizing the progression of the bolus of contrast agent in the large thoracic vessels and the myocardial compartments in the same image (Fig. 4). We used a saturation recovery gradient echo method (repetition time/echo time 3.0 ms/1.0 ms, flip angle 15°; effective k-t SENSE acceleration 3.8-fold, spatial resolution 1.2 × 1.2 × 10 mm^3^, and saturation recovery delay 120 ms). Vector-electro-cardiographic triggering was simulated at a cardiac frequency of 60 beats per minute. Data were acquired during first pass of a bolus of gadobutrol (Gadovist®, Bayer Schering, Berlin, Germany) injected at 4 mL/s followed by a 20 mL saline flush. Each bolus of gadobutrol was preceded by a diluted prebolus with 10% of the dose to allow quantification of myocardial blood flow, according to published methods (6–8). To avoid any interactions between the first and the second injection of contrast agent, a long pause was programmed on the injector to allow for a complete washout of gadolinium from the myocardial compartments between the first and the second injection. Several experimental protocols were used to assess the response of the system to isolated alterations of the myocardial perfusion rate, to different dosages of contrast agent or to alterations of the cardiac output. Furthermore, repeated acquisitions of SI curves in the same experimental conditions (*n* = 6) were carried out to test the reproducibility of the SI measurements.

### Sensitivity to Different Contrast Agent Dose

To assess the effects of different dosages of contrast agent on the SI of the AIF and to calculate the saturation ratio (expected peak SI/observed peak SI), gadobutrol was injected at 0.0005, 0.001, 0.0025, 0.005, 0.01, and 0.1 mmol/kg in the following experimental conditions: cardiac output 3 L/min, right and left myocardial perfusion rate 10 mL/mL/min. To assess the effects of different dosages of contrast agent on the SI of the myocardial compartments and to calculate the saturation ratio, gadobutrol was injected at 0.001, 0.0025, 0.005, and 0.01 mmol/kg in the following experimental conditions: cardiac output 3 L/min, right and left myocardial perfusion rate 10 mL/mL/min.

### Sensitivity to Myocardial Perfusion Rate

To assess the sensitivity of the system to different myocardial perfusion rates, first-pass perfusion measurements were performed varying the perfusion rate in the L-myoc (1, 2.5, 5, 7.5, and 10 mL/mL/min), in the following experimental conditions: cardiac output 3 L/min, contrast agent dosage 0.01 mmol/kg body weight.

### Sensitivity to Cardiac Output

To assess the effect of variations of the dilution of a bolus of contrast agent on the measured SI curves, the acquisition was performed for different values of cardiac output of 3 and 4 L/min, injecting 0.01 mmol/kg of gadolinium, with right and left myocardial perfusion rate constant at 5 mL/mL/min.

### Reproducibility Experiments

To assess the reproducibility of the measurements, two operators repeated this last group of experiments twice on different days. Moreover, reproducibility was also assessed by repeating the experiments six times under the same experimental conditions of 4 L/min of cardiac output, using 0.01 mmol/kg of gadolinium, and a perfusion rate of 1 and 5 mL/mL/min in the right and 10 mL/mL/min in the left myocardial compartment.

### Image Analysis

Data were analysed using ViewForum v6.3.1.2 (Philips) modified with software made in-house, which allows efficient segmentation of the images and export of the SI curves for analysis. Data were analysed by one of the authors who was unaware of the protocol and perfusion rate used in each experiment. Quantification of myocardial perfusion was performed using a Fermi deconvolution method (9).

Both the extracted AIF SI curve *c*_in_(*t*) and myocardial compartment SI curve *q*(*t*) values were entered into the deconvolution model that is based on the central volume principle (10, 11):


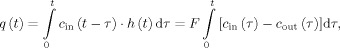


in which *F* denotes perfusion flow and *c*_out_(*t*) denote the contrast concentrations in the venous outflow. The tissue impulse response *h*(*t*) is estimated by using a Marquardt–Levenberg nonlinear least square optimization method to fit a Fermi function with the following analytical expression:


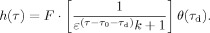


In the above equation, *F* and *k* represent indices of the contrast agent influx and efflux parameters, θ(τ_d_) is the unit step function, τ_d_ accounts for the delay time between the appearance of signal in LV blood pool *c*_in_(*t*) and myocardial region of interest *q*(*t*), and finally τ_0_ characterizes the width of the shoulder of the Fermi function during which little or no contrast agent has left region of interest. This fitting procedure yielded the time curves for tissue impulse response function, *h*(*t*), from which perfusion values were calculated as (*h*(*t* = 0)) (9).

### Statistical Analysis

SI curves were compared to assess reproducibility by means of a linear regression analysis using the Pearson's correlation coefficient. Multiple measurements were compared using the analysis of variance (ANOVA) test. All data analysis was performed with Predictive Analytics SoftWare (PASW) statistics for Mac 18.0.0 (SPSS, Chicago, IL).

## RESULTS

### Sensitivity to Different Contrast Agent Dosages

**FIG. 5:**
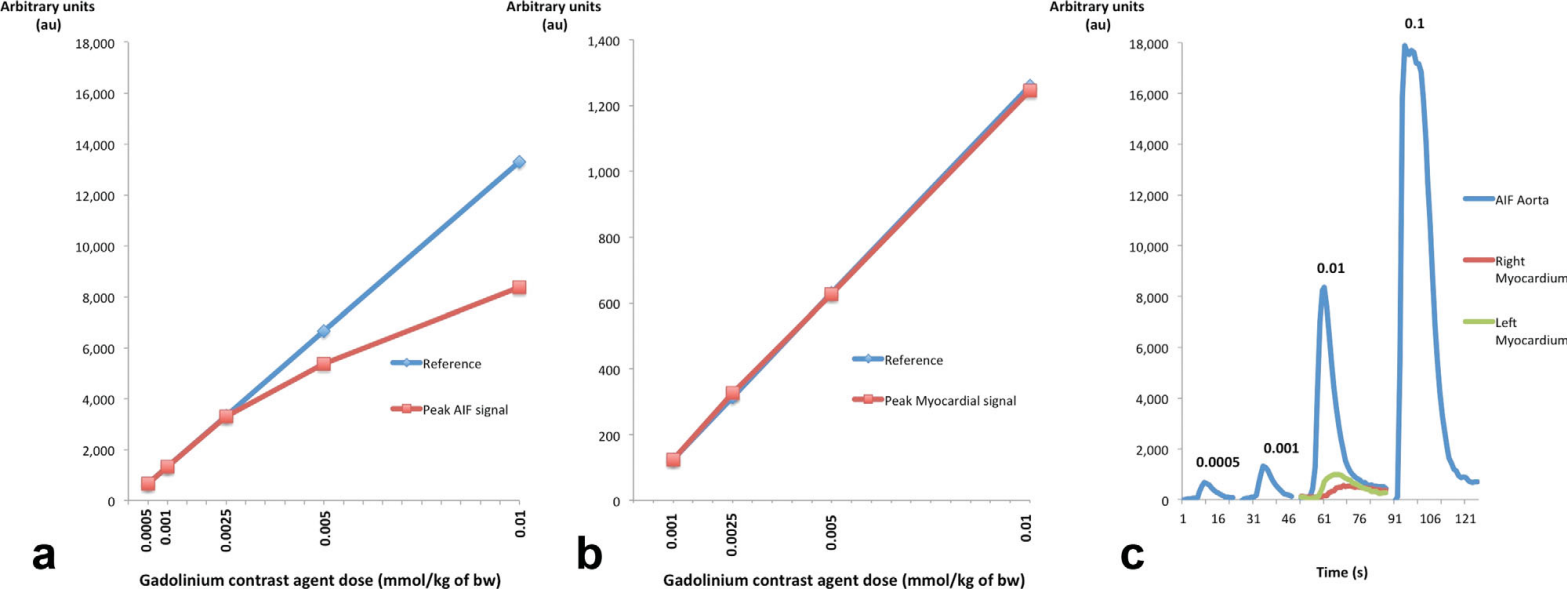
Response of the system to different dosages of contrast agent. **a**: Arterial input function peak signal intensity for different dosages of contrast agent. Dosages of 0.0005, 0.001, 0.01, and 0.1 mmol/kg of body weight injected in the system under constant experimental conditions (see text for details), producing an increasing amplitude of the AIF measured in the aorta. **b**: Myocardial peak signal intensity for different dosages of contrast agent. Dosages of 0.001, 0.0025, 0.005, and 0.01 mmol/kg of body weight injected in the system with constant myocardial perfusion rate (10 mL/mL/min). No saturation effects were observed in the range of concentrations tested. **c**: Dosages of 0.0005, 0.001, 0.01, and 0.1 mmol/kg of body weight were injected in the system under constant experimental conditions (see text for details), producing an increasing amplitude of the AIF measured in the aorta. Saturation effects with clipping of the signal intensity curve are visually observed at 0.1 mmol/kg of body weight. Myocardial SI curves are represented for the 0.01 mmol/kg of body weight injection. Right myocardium: 2.5 mL/mL/min; left myocardium 10 mL/mL/min.

A progressive increase in the peak AIF SI was noted with increasing doses of gadolinium (Fig. 5a). A very low dose of 0.0005 mmol/kg of body weight of gadolinium gave a peak AIF intensity of 665 arbitrary units (au). An injection of 0.001 mmol/kg of body weight gave a peak AIF SI of 1335 au (vs. an expected value of 1330 au), showing no saturation effects at this dosage (saturation ratio 1). An injection of 0.0025 mmol/kg of body weight gave a peak AIF SI of 3308 au (expected 3325 au), without appreciable saturation effects. At higher dosages, progressive saturation effects occurred. Injections of 0.005, 0.01, and 0.1 mmol/kg of body weight gave peak values of the AIF of 5369 au (expected 6650 au), 8365 au (expected 13300 au), and 17894 au (expected 133000 au), with saturation ratios of 1.24, 1.59, and 7.43, respectively. These findings show a very good agreement with human data available in the literature.

A progressive increase of SI in the myocardial compartments was also obtained in the myocardial compartments following an increase in the dosage of contrast agent administered (Fig. 5b). At a dosage of 0.001 mmol/kg of body weight, the myocardial peak SI was 125 au. At dosages of 0.0025, 0.005, and 0.01 mmol/kg of body weight, the myocardial peak SI was 327 au (expected 313 au), 628 au (expected 630 au), and 1245 au (expected 1260 au), respectively, with saturation ratio very close to 1 for all dosages. Moreover, Fermi deconvolution quantification of myocardial blood flow gave accurate perfusion estimated across the whole range of dosages tested (gold standard perfusion rate 10 mL/mL/min) of 9.7 ± 2.1 mL/mL/min, 9.9 ± 1.3 mL/mL/min, and 10.1 ± 1.2 mL/mL/min at 0.0025, 0.005, and 0.01 mmol/kg of body weight, respectively. To avoid any confounding effects from signal saturation, all quantitative data presented in this manuscript were obtained by deconvolving the aortic AIF (obtained after a diluted prebolus of 0.001 mmol/kg of body weight) with myocardial SI curves obtained by an injection with 0.01 mmol/kg of body weight.

### Sensitivity to Myocardial Perfusion Rate

**FIG. 6:**
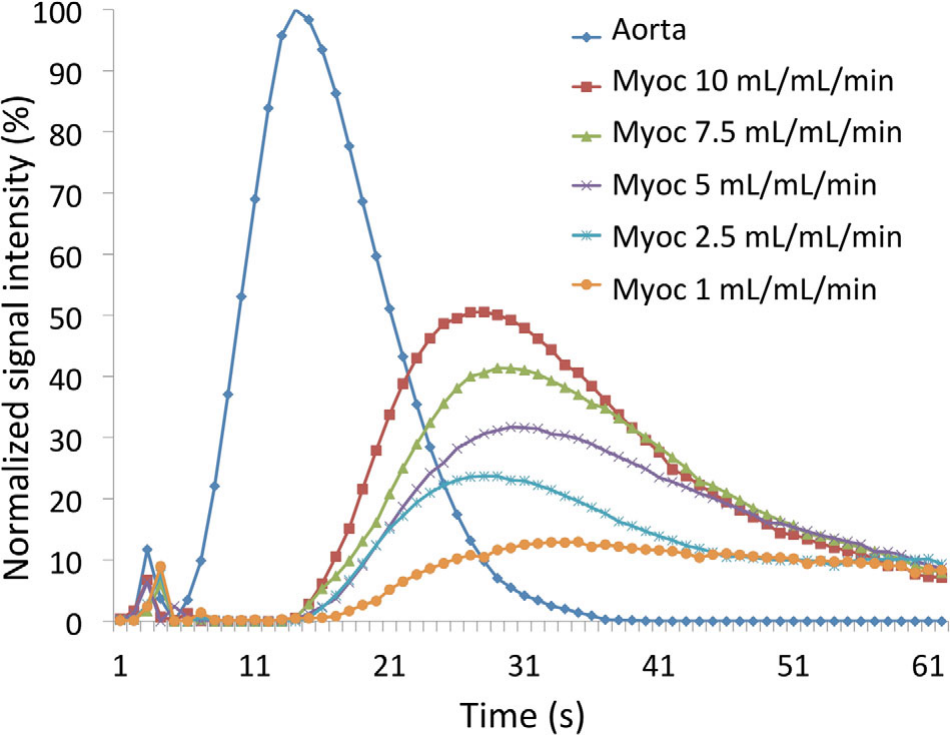
Response of the system to isolated changes of the myocardial perfusion rate. The graph represents the myocardial signal intensity curves at different perfusion rates (1, 2.5, 5, 7.5, and 10 mL/mL/min) normalized on the aortic AIF.

The system showed good sensitivity for different perfusion rates, generating independent curves for the different perfusion values tested (between 1 and 10 mL/mL/min). Figure 6 shows the time-intensity curves recorded from the aorta and the myocardial compartments for different perfusion rates.

Quantification of myocardial perfusion provided results consistent with the gold standard perfusion measurements obtained by the phantom's flow meters. The results were as follows (deconvolution measured perfusion rate ± standard deviation/actual perfusion rate): 10.4 ± 0.4/10, 7.4 ± 0.3/7.5, 4.7 ± 0.1/5, 2.9 ± 0.2/2.5, and 1.3 ± 0.4/1 mL/mL/min (*P* < 0.0001 among different flow rates; *n* = 6).

### Sensitivity to Cardiac Output

**FIG. 7:**
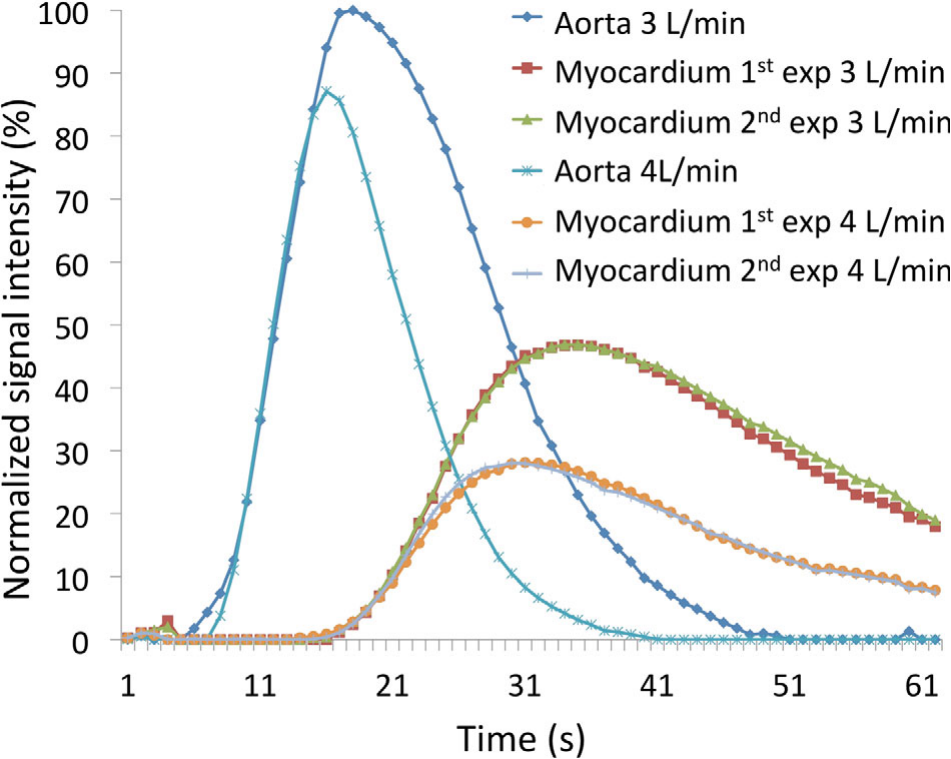
Response of the system to isolated changes of the cardiac output and reproducibility of the measurements. Each experiment was performed twice with cardiac output at 3 and 4 L/min and demonstrates the effects of different dilution rates on the peak signal intensity and speed of washout of the AIF (aorta) and in the myocardial compartment. The experiments were repeated by different operators and on different days, showing a very good reproducibility of the measurements. [Color figure can be viewed in the online issue, which is available at wileyonlinelibrary.com.]

The system also demonstrated a good response to different cardiac output rates. At 4 L/min, the system produced a shorter and lower amplitude aortic SI curve when compared with 3 L/min (Fig. 7). The higher dilution rate and faster washout associated with the higher cardiac output value produced a lower peak concentration of gadolinium in the aorta. The amplitude to the corresponding myocardial SI curves was proportional to the concentration of the contrast agent in the perfusate.

### Reproducibility Experiments

The latter experiment was repeated several times (*n* = 6), showing excellent reproducibility between different operators and on different days both for cardiac output of 3 L/min (*R*^2^ 0.999; *P* < 0.0001) and 4 L/min (*R*^2^ 0.998; *P* < 0.0001). Reproducibility was also demonstrated for different myocardial perfusion rates, as described in Methods section. The aortic, right, and left myocardial SI curves showed a very good correlation between experiments, with an adjusted *R*^2^ of 0.99 and a *P* < 0.0001 consistently.

## DISCUSSION

This study demonstrates the potential of a novel hardware MR phantom for the simulation of myocardial first-pass MR perfusion. The system allows validation of quantitative analysis versus physical measurements of flow and perfusion in different conditions of myocardial blood flow, cardiac output, and contrast agent's dosage. The system is highly reproducible and therefore allows the comparison and development of novel techniques. Moreover, the presence of two independently perfused and regulated myocardial compartments allows individual alterations to be made in the myocardial blood flow of one or both. If flow is kept constant in one compartment, it can be used as a reference standard and quality control for the acquired images while modifying the perfusion rate in the other. The use of a clinical MR scanner allows testing and development of clinical protocols, with the possibility of very quick translation of novel MR methods.

New MR sequences offer the possibility of unprecedented spatial resolution (12–15), and optimized infusion schemes and postprocessing techniques allow true quantification of myocardial perfusion in patients (4). However, the development of novel MR techniques as well as postprocessing methods is currently performed in preclinical studies using static phantoms, simulated data, or animal experiments, or in clinical trials in volunteers and patients. Our system has several advantages over the other available preclinical and clinical experimental models.

### Comparison With Simulated Data and Phantom Experiments

Synthetic data simulate the AIF and myocardial SI curves at different perfusion rates (9, 16–18). Such simulations are intended as benchmarks for deconvolution methods under controlled conditions and known simulated perfusion rates. Although extensively used in the past, these simulations lack standardization and vary from one study to another, hampering comparison of the results between different sites. Furthermore, simulated data do not completely address scanning artifacts (like saturation or susceptibility effects) and ignore spatial relations within the images. Moreover, the level of noise in the data is simulated as well. Although simulations allow isolation of the deconvolution problem, they could lead to the development of analysis methods that are not applicable to a real-world scenario. Moreover, no gold standard validation is available and the development of new sequences or novel MR hardware is precluded. To partially overcome these limitations, vials containing water and gadolinium in different concentrations have been used to acquire MR perfusion images and calculate signal-to-noise ratio and signal saturation for different *T*_1_ values of the samples (16, 19).

These methods allow the acquisition of real MR data, testing and comparing novel sequences and hardware. However, the SI curves reconstructed from the images result from simulations and quantitative results lack validation against true perfusion measurements. Finally, these static phantoms do not allow the comparison between different schemes of contrast agent injection and do not allow any simulation of the relevant physiological parameters.

Recently, a dynamic flow imaging phantom has been described to provide reproducibility assessment and validation of dynamic contrast-enhanced computed tomography (20). This system, which is potentially MR compatible, mimics realistic time attenuation curves by modulating a contrast injection pump and the ratio between the flow in the main circuit and in a compartment providing a simulation of the tissue response curve. In this study, the computed tomography flow phantom was validated using mathematical models including the control parameters of the system rather than by measuring the flow across the sections of the circuit, and the aim was to produce reproducible time attenuation curves for the comparison and assessment of the reproducibility using different computed tomography scanners. The validation of quantitative perfusion measurements was not the main purpose of the computed tomography flow phantom.

### Comparison With Animal Experiments

Animal experiments have been used to validate semiquantitative and true quantitative assessments of myocardial perfusion (6, 7, 21). These models offer realistic and physiological generation of the signal and allow invasive procedures, such as microspheres injection, for validation of the results. However, the high costs and ethical and logistic considerations limit their applicability.

To overcome these limitations in part, some novel preclinical models have been recently developed within our group. Makowski et al. has described a method of performing first-pass MR perfusion imaging in rodents (22), using the k-t principal component analysis techniques (23) and a clinical 3T MR scanner. The availability of many transgenic models of cardiovascular disease makes this method particularly attractive.

Schuster et al. have also described a novel explanted and blood perfused pig heart MR compatible model to develop and validate perfusion acquisitions (24). This model offers much greater control over physiological parameters and better reproducibility compared with in vivo preparations, although it is less physiological. This isolated pig heart model can be studied in a clinical scanner. In addition, the porcine heart is of comparable dimensions to a human heart. These factors facilitate the development, validation, and translation of new perfusion methods. However, operating this experimental model in a clinical scanner is associated with higher costs and requires considerable preparation times, and should thus be restricted to the validation of predeveloped methodology.

### Comparison With Human Studies

Human studies should in theory offer the best setup for the validation of novel MR perfusion methods. Although several studies have been performed comparing the diagnostic accuracy of MR perfusion with coronary angiography and fractional flow reserve assessment (25, 26), the validation of quantitative perfusion assessment can only be performed by comparing MR imaging with positron emission tomography (27–29). Several drawbacks limit these studies, including the lack of a direct real validation, the costs of the procedure, and some ethical concerns due to the use of ionizing radiation. As a result, clinical trials are best reserved for comparative and outcome studies.

Studies using the perfusion phantom have different aims and will not compete with clinical studies, as the phantom does not allow outcome studies or comparison of the diagnostic accuracy of MR perfusion in the detection of coronary artery disease. It was designed to allow reproducible and realistic simulation of first-pass perfusion and to offer true validation of the results of quantitative analysis without the need for lengthy and expensive laboratory analyses. The phantom is cheaper than the competing solutions, all of the equipment can be reused, and the acquisition process is very efficient, as washout of the contrast in the open circuit model only requires 60–180 s (depending on the set perfusion rate) before a new perfusion experiment can be performed. Our data demonstrate that the perfusion phantom provides data suitable for quantification by means of signal deconvolution. Future work will be needed to validate existing analysis methods and to develop and validate novel approaches toward full quantification. However, the phantom is likely to allow a reduction of the number of animal studies required to develop and validate novel MR techniques.

## LIMITATIONS OF THE STUDY

The main limitation of the current setup of the perfusion phantom is that the distribution dynamics of the contrast agent in the myocardial compartments only simulates the physiological appearance of the SI curves and does not involve diffusion of the contrast from the vascular to the interstitial space (see Fig. 3) as occurs in vivo. Therefore, the myocardial space acts as a single compartment for the distribution of gadolinium.

There are some other differences between the myocardial compartments of the phantom and the real myocardial tissue. The average resistance to blood flow in the synthetic myocardium is lower than the physiological one and does not vary over time as result of the complex interaction between cardiac contraction and small intramyocardial vessels. Moreover, the myocardial compartments show a relatively narrow distribution and a lower incoherence of flow compared with the myocardial capillary bed. For this reasons, we feel that the perfusion phantom cannot completely replace animal experiments or human validation studies. However, the capability of the perfusion phantom to offer a controlled and highly reproducible simulation of first-pass perfusion, with selective alterations of myocardial blood flow in one or both myocardial compartments, is likely to expedite the development and comparison of different acquisition sequences or hardware or direct comparison of different quantification techniques, which is very difficult to achieve in vivo.

A possible confounding effect in first-pass perfusion quantification is due to bolus dispersion that occurs during the transit through the epicardial vessels to the myocardium (5). Even though dispersion effects cannot be completely excluded, these are likely to play a minor role in the current setup of the phantom due to: (1) the physiological design and size of the coronaries and myocardial compartments of the phantom, resulting in physiological flow and perfusion rates; (2) the fixed geometry of the coronaries (alterations of the perfusion rate are generated by changing the speed of the roller pumps downstream the imaging plane and not by alterations of the vascular geometry-stenosis); (3) the use of a continuous perfusion flow, eliminating the risk for temporal variations of dispersion due to the reflection of pressure waves (30). Moreover, we observed a very good correspondence between the gold standard perfusion rate measurements and deconvolution-based perfusion measurements throughout the range of perfusion rates tested (1–10 mL/mL/min). Although minimized by the current setup, bolus dispersion should be considered among the possible reasons for discrepancies between phantom studies and in vivo studies.

Other limitations of the setup presented are intrinsic to the methods currently used to acquire first-pass perfusion images. Turbo gradient-echo sequences cause an acceleration of *T*_1_ relaxation leading to an apparent *T*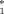. Moreover, during first-pass perfusion experiments, different relaxation rates in different compartments and spin diffusion phenomena further affect the reliability of the detected SI (31, 32). These limitations, which are also applicable to any other experimental model currently in use and equally apply to clinical studies, need to be considered while evaluating the results.

The use of Fermi deconvolution is meant, in this study, as a means to demonstrate that the perfusion phantom is capable, in the described setup, to provide realistic simulations of myocardial perfusion that are suitable for quantification by means of deconvolution algorithms. We have decided to apply Fermi deconvolution, instead of any of the other mathematical methods previously described in the literature, as it has been extensively used in the past to quantify myocardial perfusion. However, the validation of Fermi deconvolution or any other deconvolution method is outside the aim of this article and specific comparative studies will be required to compare different deconvolution algorithms and to define the most accurate quantification method.

## CONCLUSIONS

This novel hardware perfusion phantom allows reliable, reproducible, and efficient simulation of myocardial perfusion. The availability of a direct comparison between the image data and reference values of flow and perfusion will allow rapid development and validation of accurate quantification methods.
